# Traumatic dental injuries in the primary dentition: exploratory medico-legal framework for temporary damage assessment

**DOI:** 10.1007/s00414-026-03918-7

**Published:** 2026-07-15

**Authors:** Patrizia Defabianis, Francesca Beducci, Emilio Nuzzolese

**Affiliations:** 1 School of Pediatric Dentistry, Department of Surgical Sciences, University of Turin, Turin, Italy; 2 Section of Legal Medicine, Department of Public Health and Pediatric Sciences, University of Turin, Turin, Italy

**Keywords:** Traumatic dental injuries, Primary dentition, Forensic appraisal, Temporary biological damage, Paediatric dental trauma, Forensic odontology

## Abstract

Traumatic dental injuries (TDIs) in the primary dentition are frequent in early childhood and may lead to sequelae affecting both injured primary teeth and their permanent successors. They also raise medico-legal questions when premature loss of a primary tooth results in a time-dependent functional, aesthetic, and psychosocial impairment before the permanent successor can be evaluated. This retrospective single-center study reviewed the records of 419 children (262 males, 157 females; mean age 3.23 ± 1.85 years) treated for TDIs to the primary dentition between 2013 and 2022, comprising 428 traumatic events and 780 affected primary teeth. Demographic data, injury circumstances, trauma type, affected teeth, treatment, follow-up, and sequelae were analysed at the patient, event, and tooth levels. Luxation injuries predominated, domestic accidents were the most frequent circumstance of injury, and maxillary central incisors were the most commonly affected teeth. Pulp necrosis was recorded in 14.3% of injured teeth, delayed premature loss in 21.0%, and permanent successor sequelae in 19.0% based on available follow-up records. The secondary aim was medico-legal: to inform an exploratory framework for assessing temporary biological damage during the interval between premature tooth loss and eruption of the permanent successor. The proposed framework is not presented as a validated compensation scale but as a conceptual aid for expert appraisal. Prospective multicenter validation is required before any normative or compensation use.

## Introduction

Traumatic dental injuries (TDIs) constitute a frequent public health issue with significant functional and psychosocial repercussions that adversely affect quality of life [1]. They account for approximately 5% of all injuries among children and young adults [2]. Epidemiological data indicate that around 17% of preschool children, 25% of school-age children, and up to 33% of adults experience at least one TDI, most occurring before age 19 [3]. A recent systematic review and meta-analysis estimated the prevalence of trauma in the primary dentition at approximately 22.7% [4], and longitudinal evidence indicates that a considerable subset of children sustain multiple events over time [5, 6]. Because minor injuries are often undocumented, the true epidemiological burden is likely to be underestimated [7].

Early childhood is particularly vulnerable. Developing psychomotor skills, combined with early activities such as crawling, learning to walk, and play-related collisions, increase the risk of falls and facial impacts [8]. Motor incoordination, hyperactivity, and limited inhibitory control further predispose young children to orofacial trauma involving the primary dentition.

Injuries to primary teeth can cause immediate or delayed sequelae such as pulp necrosis (with or without abscess or fistula), premature tooth loss, gingival recession, crown discolouration, pathological root resorption, root canal obliteration, and loss of arch space [9–11]. Of particular concern are sequelae affecting permanent successors, manifesting as enamel defects, eruption delay or displacement, crown or root dilaceration, odontoma-like malformations, or even sequestration of the tooth germ [12–15]. The reported prevalence of such outcomes ranges from 12% to 74% [12, 16].

Intrusive luxations and avulsions are the injury types most associated with permanent developmental disturbances [12, 13, 17]. The link is explained by the close anatomical proximity of the primary root apex to the developing permanent germ [18–21]. Beyond structural implications, traumatic alteration or loss of primary teeth can have functional, aesthetic, psychological, and social ramifications for the child and family [22–29].

The present study had a dual aim. First, to describe the epidemiology, clinical characteristics, management, and sequelae of traumatic dental injuries affecting the primary dentition in a university-based cohort. Second, to use these observations to inform an exploratory medico-legal framework for assessing temporary biological damage during the interval between premature loss of a primary tooth and eruption of the permanent successor. The study was not designed to determine fault allocation, negligence, or a legally binding compensation tariff. Rather, it seeks to identify clinically grounded parameters relevant to forensic appraisal in paediatric dental trauma.

## Materials and methods

This study was reported in accordance with the Strengthening the Reporting of Observational Studies in Epidemiology (STROBE) guidelines for cross-sectional studies [30]. This retrospective single-center observational study was conducted at the Department of Paediatric Dentistry, University of Turin, Turin, Italy, and included children aged 0–11 years who were treated for traumatic injuries to the primary dentition between January 2013 and December 2022. Records of all children who presented with primary tooth trauma during the study period were reviewed. Cases involving pre-existing developmental defects, caries-related damage, or incomplete documentation were excluded; the exact number of excluded records was not documented. The final study sample comprised 419 patients (262 males and 157 females), accounting for 428 traumatic events and 780 affected primary teeth; unless otherwise specified, percentages refer to affected teeth.

The study was based on anonymised clinical records and was conducted in accordance with institutional regulations and the General Data Protection Regulation (EU 2016/679). Ethical review was waived by the Institutional Review Board of the University of Turin because of the retrospective, non-interventional design and the use of anonymised data. Written informed consent for clinical care and secondary use of anonymised data had been obtained from the legal guardians.

The following variables were recorded: sex, age at trauma, year of injury, place and mechanism of injury, delay to first examination, injury type, affected teeth, associated soft-tissue injuries, treatment rendered, follow-up duration, sequelae affecting the primary dentition, and sequelae affecting the permanent successor when assessable. Injuries were classified according to the WHO classification modified by Andreasen [15]. Exclusion criteria were pre-existing dental defects, carious lesions involving the injured teeth, and cases with follow-up shorter than 2 months in the absence of complications. Premature loss was defined as spontaneous loss or extraction of a traumatised primary tooth during follow-up before physiological exfoliation. Teeth lost at the time of trauma by avulsion were considered separately and were not included in the analysis of delayed premature loss. Permanent sequelae were recorded only when the permanent successor could be assessed clinically and/or radiographically.

Patients were stratified into 2 age groups according to age at trauma: younger than 4 years (< 4 years) and 4 years or older (≥ 4 years). This cut-off was chosen because the developmental stage of the permanent successor and the clinical setting of traumatic events differ meaningfully across this threshold, with younger children more frequently sustaining trauma in the domestic environment and at earlier stages of permanent tooth mineralisation.

Because the study period extended across publication of the 2020 International Association of Dental Traumatology (IADT) recommendations [5], cases were also grouped into a pre-2020 period and a post-2020 period to explore possible temporal heterogeneity in management and outcome ascertainment. Diagnostic classification remained based on the WHO/Andreasen framework throughout the study period, whereas clinical management reflected the departmental protocol in force at the time of treatment.

Follow-up duration was summarised using the median and range; the median follow-up was 30 months (range, 12–84 months). Statistical analysis was performed using IBM SPSS Statistics, version 26.0 (IBM Corp., Armonk, NY, USA). Analyses were planned at three levels: patient, traumatic event, and tooth. Patient-level variables included sex and age at trauma; event-level variables included year, place and mechanism of injury, and delay to first examination; tooth-level variables included tooth type, injury type, treatment, and sequelae. Descriptive statistics were used to summarise demographic, clinical, and injury-related characteristics. Continuous variables are reported as mean and standard deviation or median and interquartile range, as appropriate, whereas categorical variables are reported as absolute frequencies and percentages. Exact binomial 95% confidence intervals (CIs) were calculated for the principal prevalence estimates. Associations between trauma type and selected sequelae were explored using univariate chi-square tests, each comparing a given trauma category against all remaining categories combined (2 × 2 contingency tables, df = 1). No adjustment for multiple comparisons was applied; results should therefore be interpreted as exploratory. All tests were two-sided, and statistical significance was set at *p* < 0.05. The unit of analysis varied according to the variable studied: demographic characteristics were reported at the patient level, injury circumstances at the traumatic-event level, and injury type, complications, and permanent successor sequelae at the tooth level. Because multiple teeth could be involved in the same patient, tooth-level analyses did not account for within-patient clustering and should be interpreted accordingly.

The medico-legal component comprised an exploratory framework for temporary biological damage during the interval between traumatic loss of a primary tooth and eruption of the permanent successor. The proposed coefficients were derived by proportional adaptation of established medico-legal reference values for the permanent dentition [31, 32], scaled to the deciduous hemiarch and distributed according to the relative functional and anatomical relevance of each tooth. When a functional space maintainer with prosthetic tooth replacement was used, a one-third reduction of the corresponding coefficient was assumed to reflect partial restoration of function and aesthetics. This framework was not empirically calibrated and should be interpreted as a structured aid for expert appraisal only.

## Results

**Fig. 1:**
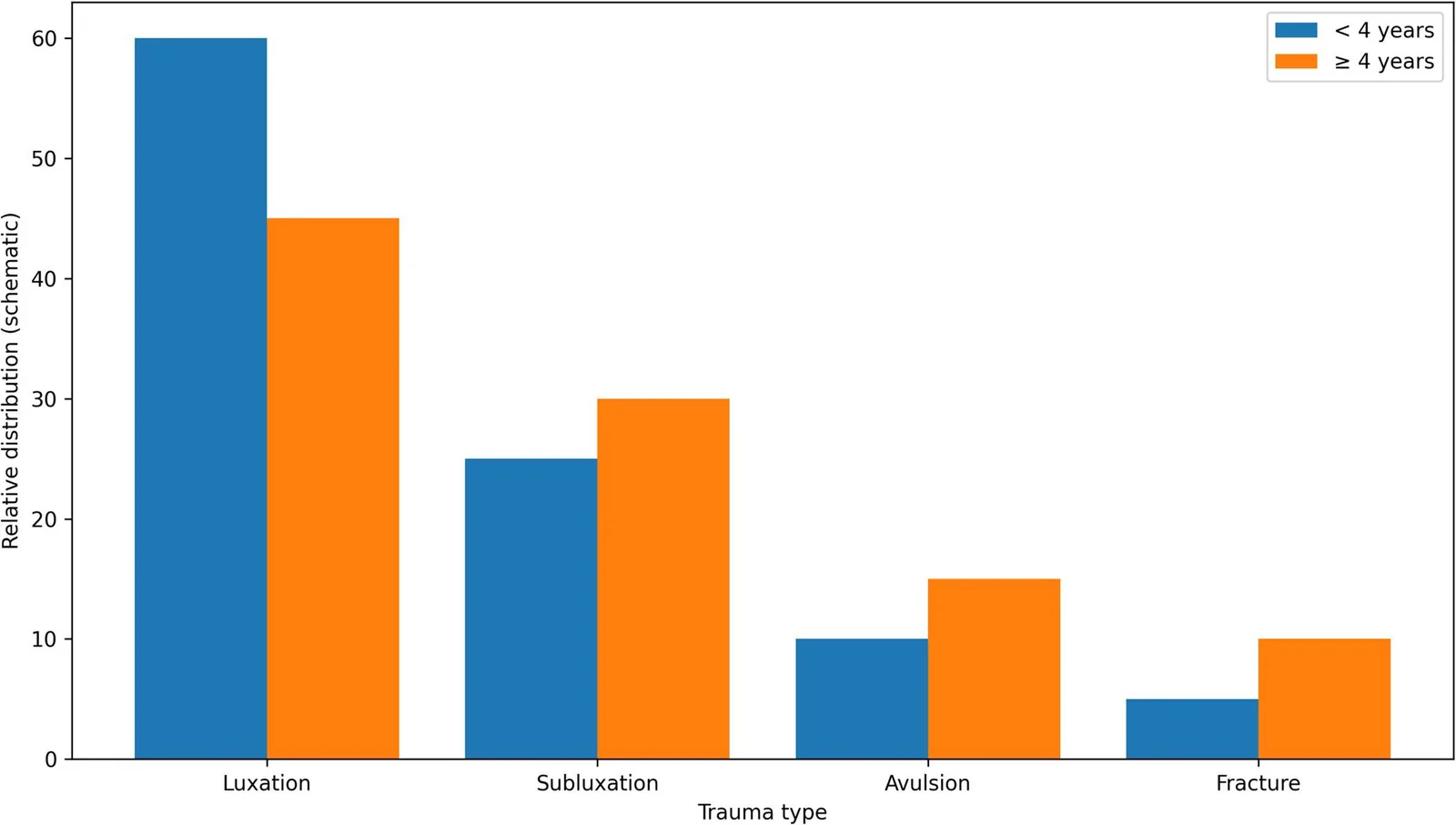
Distribution of trauma type and sequelae by age group

The sample comprised 419 patients (262 males, 62.5%; 157 females, 37.5%), with a mean age at trauma of 3.23 ± 1.85 years. A total of 428 traumatic events were recorded, involving 780 primary teeth. Most traumatic events occurred in domestic settings, followed by school-related and recreation-related accidents (Fig. 1).

Among the 780 affected primary teeth, the maxillary central incisors were the teeth most frequently involved (569/780, 73.0%), followed by the maxillary lateral incisors (140/780, 18.0%).

Subluxation was the most frequent injury type, followed by luxation, crown fracture, avulsion, and concussion. Associated soft-tissue injuries, mainly involving the gingiva, were observed in nearly two thirds of patients.

Most traumatised primary teeth healed without clinically relevant complications. However, pulp necrosis was recorded in 112/780 teeth (14.3%, 95% CI 12.0–17.0), crown discolouration in 92/780 (11.8%, 95% CI 9.6–14.3), and delayed premature loss in 164/780 (21.0%, 95% CI 18.2–24.1). Luxation injuries were significantly associated with delayed premature loss (χ² = 4.62, df = 1, *p* = 0.032).

With regard to the permanent dentition, permanent successor sequelae were documented in 148 of 780 injured teeth (19.0%, 95% CI 16.3–21.9), based on the available clinical and/or radiographic follow-up records. However, this denominator includes all injured teeth regardless of whether follow-up was sufficient for clinical and/or radiographic assessment of the permanent successor. The reported prevalence should therefore be regarded as a minimum estimate.

The most frequent permanent successor sequelae were delayed eruption, ectopic eruption, and enamel defects. When stratified by trauma type, avulsion showed the highest proportion of permanent developmental disturbances (29/46, 63.0%) and was significantly associated with sequelae in the permanent successor (χ² = 9.11, df = 1, *p* < 0.01). Fracture injuries showed a lower and non-significant rate of successor sequelae (6/126, 4.8%; χ² = 1.02, df = 1, *p* = 0.31). These associations are summarised in Table 1.

Children younger than 4 years showed a higher relative frequency of luxation injuries and of permanent successor disturbances than children aged 4 years or older, supporting the clinical relevance of age at trauma in the interpretation of both sequelae and medico-legal appraisal.

Cases were additionally compared between the pre-2020 period and the post-2020 period following publication of the 2020 IADT recommendations [5]. No substantial differences were observed in the distribution of trauma types, major sequelae, or follow-up outcomes between the two periods. Given the retrospective design and the limited size of the period-specific subgroups, these comparisons were considered exploratory.

**Table 1:** Association between selected trauma types, delayed premature loss, and permanent successor sequelae

Trauma type	Delayed premature loss, *n* (%)	Permanent successor sequelae, *n* (%)	χ² (df)	*p*-value
Luxation	84 (28.4%)	32 (10.8%)	4.62 (1)^a	0.032
Avulsion	NA	29 (63.0%)	9.11 (1)^b	< 0.01
Fracture	12 (9.5%)	6 (4.8%)	1.02 (1)^b	0.31

^a Test refers to delayed premature loss. ^b Test refers to permanent successor sequelae. Each trauma type was compared against all other types combined (2 × 2 contingency table). Delayed premature loss was defined as spontaneous loss or extraction during follow-up before physiological exfoliation; teeth lost at the time of trauma by avulsion were therefore not included in this outcome. Permanent successor sequelae are reported over the full cohort of 780 injured teeth, based on the available clinical and/or radiographic follow-up records. No adjustment for multiple comparisons was applied

## Discussion

This decade-long study confirms that traumatic dental injuries affecting the primary dentition occur predominantly in boys younger than 4 years of age and are most commonly associated with domestic accidents, in line with previous reports [3, 7, 8]. Luxation injuries were the most frequent trauma type, consistent with the anatomical and biomechanical characteristics of primary teeth and their supporting tissues [15, 33]. The predominance of the maxillary central incisors is a well-established finding across populations and reflects the anatomical exposure of these teeth to frontal impact [4, 11]. Permanent successor disturbances were documented in a clinically relevant proportion of cases, and their association with avulsion is consistent with previous longitudinal studies [12–14]. As this was a single-center Italian cohort, the observed distribution of injury mechanisms and outcomes may reflect local demographic and referral characteristics and should therefore be interpreted with caution when extrapolating to other populations.

Pulp necrosis (14.3%), crown discoloration (11.8%), and delayed premature loss (21.0%) were the most commonly recorded complications in the primary dentition. The significant association between luxation injuries and delayed premature loss is consistent with the biomechanical disruption of the periodontal ligament characteristic of these injuries. The permanent successor sequelae rate of 19.0% should be interpreted with an important caveat: because the median follow-up was 30 months and the permanent central incisors typically erupt at age 6–7 years, many children traumatised at young ages had not yet reached the stage at which successor outcomes could be assessed. The true proportion of successor disturbances may therefore be higher than reported here, and late-developing sequelae may be underrepresented. Future studies with longer and more uniform follow-up will be needed to obtain more precise estimates.

The present dataset provides clinically relevant information on injury patterns, treatment, and sequelae, but it does not by itself validate a compensation schedule. For this reason, the medico-legal component of the manuscript should be understood as an exploratory appraisal framework intended to support structured expert reasoning in cases involving premature loss of a primary tooth. The study addresses damage assessment after a documented traumatic event; it does not address fault allocation or negligence. Its relevance lies in the interval between premature loss of the primary tooth and clinical assessment of the permanent successor, a period during which functional, aesthetic, and psychosocial consequences may be present but remain time-dependent and individually variable.

A central medico-legal difficulty in these cases is that definitive permanent damage can be assessed only after eruption of the permanent successor, whereas the child may experience a prolonged interim period marked by aesthetic, phonetic, and functional impairment. Existing medico-legal reference systems—including the Italian barème system [31, 32, 34], the French barème indicatif, Nordic compensation models, and the United Kingdom Criminal Injuries Compensation Authority (CICA) tables—are calibrated for the permanent dentition and do not specifically quantify this transitional period in the primary dentition. The proposed framework was developed to address that gap in a structured, explicitly exploratory manner.

In medico-legal terms, temporary biological damage (TBD) may be understood as a time-bound impairment that does not necessarily produce a permanent anatomical defect but may still result in measurable functional, aesthetic, or psychosocial limitation [31, 32]. Although premature loss of primary incisors rarely causes major arch modification [35, 36], it may interfere with speech, feeding, appearance, and social interaction during critical developmental stages [7, 22–24]. These transient but clinically relevant limitations warrant structured consideration within forensic appraisal frameworks. More broadly, this concept parallels the notion of temporary partial disablement used in other civil compensation systems, thereby supporting its cross-disciplinary relevance.

Although the present study did not include specific child-reported outcome measures, validated quality-of-life instruments such as the Parental-Caregiver Perception Questionnaire (P-CPQ) and the Family Impact Scale (FIS) may offer useful approaches for future quantification of psychosocial burden in paediatric dental trauma and may help refine medico-legal assessment.

To address the gap identified above, a table of indicative coefficients was developed for the appraisal of TBD associated with premature loss of primary teeth (Table 2). These coefficients are proposed exclusively for conceptual forensic reasoning and are not intended as direct parameters for judicial or insurance quantification.

The derivation proceeded as follows. According to Norelli et al. [16], permanent biological damage is estimated at 12% for loss of an entire maxillary permanent hemiarch and 10% for a mandibular hemiarch. Because the primary dentition comprises only 5 teeth per hemiarch and serves a transitional rather than definitive functional role, the permanent dentition hemiarch values were scaled proportionally by the ratio 5:8, yielding reference values of 7.50% for the maxillary primary hemiarch and 6.25% for the mandibular primary hemiarch. These hemiarch totals were then distributed across the five tooth types according to their relative contribution to mastication, arch integrity, phonetic support, and aesthetic function. Among individual tooth types, the upper central incisor and upper canine both received the highest coefficient (1.75%) but for different reasons: the former primarily because of its dominant aesthetic role and phonetic contribution, the latter because of its guidance function during lateral excursion and its role in maintaining anterior–posterior arch continuity. The upper and lower second primary molars were also assigned high values (1.75% and 1.25%, respectively) given their importance as posterior occlusal anchors and space maintainers. Conversely, the lower central incisor received the lowest coefficient (0.75%), reflecting its small crown dimensions, limited aesthetic visibility, and minimal contribution to masticatory efficiency. The resulting coefficients should be regarded as preliminary heuristic estimates rather than empirically validated values. If functional substitution is provided—for example, through a removable appliance with prosthetic tooth replacement—that value may reasonably be reduced by one third, reflecting partial restoration of masticatory function, phonetic support, and aesthetic appearance. The one-third reduction was adopted as a conservative estimate; it acknowledges that current paediatric prosthetic appliances restore function and appearance incompletely, particularly in very young children with limited compliance. This value is not empirically derived and requires validation. Final reassessment after eruption of the permanent successor remains essential in order to determine whether permanent consequences are present, including developmental anomalies or absence of the successor tooth.

Although the framework is conceptually consistent with the Italian medico-legal tradition [31, 32, 34], approaches to dental damage appraisal vary internationally. Current European medico-legal debate increasingly highlights the need for shared criteria. Accordingly, the present proposal should be viewed as a contribution toward future harmonisation of paediatric dental trauma appraisal rather than as a normative or legally binding standard.

No major temporal differences emerged between the pre-2020 and post-2020 periods; however, these comparisons should be interpreted cautiously in light of the retrospective design and the limited subgroup sizes.

This study has several limitations. Its retrospective design and single-center setting limit generalisability. Follow-up was not uniform across all cases: teeth with more complete follow-up may have been more likely to have successor outcomes documented, whereas late sequelae may have been underestimated in patients lost to follow-up, introducing a potential surveillance bias in the assessment of permanent successor disturbances. Furthermore, the denominator used for calculating the prevalence of permanent successor sequelae (780 teeth) includes teeth for which follow-up may have been too short or radiographic documentation too limited to detect successor involvement. Teeth with complications may also have been more likely to return for follow-up, introducing potential selection bias. The true prevalence of permanent successor disturbances may therefore be higher than the 19.0% reported here, and this figure should be interpreted with caution. Accordingly, the reported statistical associations should be interpreted as exploratory rather than confirmatory.

The proposed TBD coefficients were extrapolated from existing medico-legal models rather than derived from direct empirical measurement, and the one-third correction factor for space maintainers is a heuristic estimate. In addition, the dataset was not specifically designed to quantify functional, psychosocial, or quality-of-life burden through validated instruments.

Future studies should pursue prospective multicenter validation and incorporate clinical, psychological, and quality-of-life outcomes, ideally using validated instruments such as the P-CPQ and FIS. A formal expert consensus process—such as a Delphi methodology involving forensic odontologists, paediatric dentists, and medico-legal specialists—would further strengthen the evidence base and facilitate adoption of a standardised framework. Such work will be necessary to refine the proposed coefficients and to move toward more harmonised medico-legal standards for appraisal of paediatric dental trauma.

**Table 2:** Exploratory medico-legal framework for appraisal of temporary biological damage following premature loss of primary teeth (conceptual proposal; not derived empirically from the present cohort and not intended as a compensation tariff)

Primary tooth	Upper arch (%)	Lower arch (%)
Central incisor	1.75	0.75
Lateral incisor	1.25	1.25
Canine	1.75	1.50
First molar	1.50	1.50
Second molar	1.25	1.25
Hemiarch total	7.50	6.25

For expert reference only; not validated for judicial or insurance quantification

The proposed values are expert-derived conceptual extrapolations informed by permanent-dentition medico-legal models [31, 32] and by the clinical rationale discussed in the text. Hemiarch totals were obtained by scaling the corresponding permanent-dentition hemiarch values (12% maxillary, 10% mandibular) by the ratio 5:8 to reflect the smaller number of teeth in the primary hemiarch. Individual tooth values were distributed according to the relative functional and aesthetic contribution of each tooth type. When a functional space maintainer with prosthetic replacement is in place, a one-third reduction of the corresponding coefficient is proposed to reflect partial restoration of function and aesthetics. These coefficients are intended solely to support structured forensic reasoning and must not be used as direct judicial, insurance, or clinical valuation parameters without empirical validation.

## Conclusions

Traumatic injuries to the primary dentition carry significant clinical and medico-legal relevance. Although long-term outcomes involving the permanent successors cannot be established immediately, medico-legal appraisal of temporary functional, aesthetic, and psychosocial impairment should not be automatically postponed until eruption of the permanent successor.

The present study proposes an exploratory medico-legal framework to estimate temporary biological damage during the period between premature primary tooth loss and eruption of the successor tooth. The derivation of the proposed coefficients—based on proportional scaling from established permanent-dentition reference values and distributed according to the functional and aesthetic contribution of each primary tooth type—has been presented transparently so that the rationale can be evaluated, critiqued, and refined by the forensic and paediatric dentistry communities. The framework is intended as a structured and pragmatic aid for forensic professionals involved in the assessment of paediatric dental trauma.

Its use remains exploratory until supported by multicenter validation and broader interdisciplinary consensus. Nonetheless, the framework highlights the need for more consistent international criteria in medico-legal appraisal of traumatic injuries to the primary dentition.

## References

[1] Richards D (2018) One billion people have experienced a traumatic dental injury. Evid Based Dent 19:34–35. 10.1038/sj.ebd.6401291 29930374

[2] Petti S, Glendor U, Andersson L (2018) World traumatic dental injury prevalence and incidence: a meta-analysis. Dent Traumatol 34:71–86. 10.1111/edt.12389 29455471

[3] Glendor U (2008) Epidemiology of traumatic dental injuries: a 12-year review of the literature. Dent Traumatol 24:603–611. 10.1111/j.1600-9657.2008.00621.x 19021651

[4] Patnana AK, Chugh A, Chugh VK, Kumar P, Vanga NRV, Singh S (2021) The prevalence of traumatic dental injuries in primary teeth: a systematic review and meta-analysis. Dent Traumatol 37:383–399 10.1111/edt.12640 33289328

[5] Day PF, Flores MT, O’Connell AC, Abbott PV, Tsilingaridis G, Fouad AF, Cohenca N, Lauridsen E, Bourguignon C, Hicks L, Andreasen JO, Cehreli ZC, Harlamb S, Kahler B, Oginni A, Semper M, Levin L (2020) International Association of Dental Traumatology guidelines for the management of traumatic dental injuries: 3. Injuries in the primary dentition. Dent Traumatol 36:343–359. 10.1111/edt.12576 32458553

[6] Goettems ML, Brancher LC, da Costa CT, Bonow MLM, Romano AR (2017) Does dental trauma in the primary dentition increase the likelihood of trauma in the permanent dentition? A longitudinal study. Clin Oral Investig 21:2415–2420 10.1007/s00784-016-2037-3 28032199

[7] Lam R (2016) Epidemiology and outcomes of traumatic dental injuries: a review of the literature. Aust Dent J 61(Suppl 1):4–20. 10.1111/adj.12395 26923445

[8] Chan YM, Williams S, Davidson LE, Drummond BK (2011) Orofacial and dental trauma of young children in Dunedin, New Zealand. Dent Traumatol 27:199–202. 10.1111/j.1600-9657.2011.00984.x 21457187

[9] Borum MK, Andreasen JO (1998) Sequelae of trauma to primary maxillary incisors. I. Complications in the primary dentition. Endod Dent Traumatol 14:31–44 10.1111/j.1600-9657.1998.tb00806.x 9643176

[10] Holan G (2004) Development of clinical and radiographic signs associated with dark discolored primary incisors following traumatic injuries: a prospective controlled study. Dent Traumatol 20:276–287 10.1111/j.1600-9657.2004.00285.x 15355387

[11] Andrade MRTC, Americano GCA, da Costa MP, Lenzi MM, Dede Waele SouchoisMarsillac M, Campos V (2021) Traumatic injuries in primary dentition and their immediate and long-term consequences: a 10-year retrospective study from the State University of Rio de Janeiro, Brazil. Eur Arch Paediatr Dent 22(6):1067–1076. 10.1007/s40368-021-00652-z 34313973

[12] do Espírito Santo Jacomo DR, Campos V (2009) Prevalence of sequelae in the permanent anterior teeth after trauma in their predecessors: a longitudinal study of 8 years. Dent Traumatol 25:300–304 10.1111/j.1600-9657.2009.00764.x 19302202

[13] De Amorim LFG, Estrela C, Costa LRRS (2011) Effects of traumatic dental injuries to primary teeth on permanent teeth: a clinical follow-up study. Dent Traumatol 27:117–121 10.1111/j.1600-9657.2010.00959.x 21199335

[14] Scerri E, Gatt G, Camilleri S, Mupparapu M (2010) Morphologic and developmental disturbances of permanent teeth following trauma to primary dentition. Quintessence Int 41:717–724 20806095

[15] Andreasen JO, Andreasen FM, Andersson L (2018) Textbook and Color Atlas of Traumatic Injuries to the Teeth, 5th edn. Wiley-Blackwell, Oxford

[16] Lenzi MM, Alexandria AK, Ferreira DM et al (2014) Does trauma in the primary dentition cause sequelae in permanent successors? A systematic review. Dent Traumatol 31:79–88 10.1111/edt.12149 25382149

[17] Assunção LRS, Ferelle A, Iwakura MLH, Cunha RF (2009) Effects on permanent teeth after luxation injuries to the primary predecessors. Dent Traumatol 25:165–170 10.1111/j.1600-9657.2008.00759.x 19290894

[18] Diab M, Elbadrawy HE (2000) Intrusion injuries of primary incisors. Part III: Effects on the permanent successors. Quintessence Int 31:377–384 11203953

[19] Von Arx T (1993) Developmental disturbances of permanent teeth following trauma to the primary dentition. Aust Dent J 38:1–10 10.1111/j.1834-7819.1993.tb05444.x 8447765

[20] Ben-Bassat Y, Brin I, Fuks A, Zilberman Y (1985) Effect of trauma to the primary incisors on their permanent successors in different developmental stages. Pediatr Dent 7:37–40 3857558

[21] Smith RJ, Rapp RA (1980) Cephalometric study of the developmental relationship between primary and permanent maxillary central incisors. J Dent Child 47:36–41 6928134

[22] Bani M, Alacam A, Cinar C (2017) How does dental trauma affect the quality of life in Turkish families? Oral Health Prev Dent 15:563–567 10.3290/j.ohpd.a38573 28681048

[23] Lee JY, Divaris K (2009) Hidden consequences of dental trauma: the social and psychological effects. Pediatr Dent 31:96–101 19455926

[24] Nadelman P, Bedran N, Magno MB, Masterson D, de Castro ACR, Maia LC (2020) Premature loss of primary anterior teeth and its consequences to primary dental arch and speech pattern: a systematic review and meta-analysis. Int J Paediatr Dent 30:687–712. 10.1111/ipd.12644 32243000

[25] Kapur A, Chawla HS, Goyal A, Gaube K (2005) An esthetic point of view in very young children. J Clin Pediatr Dent 30:99–103 16491961

[26] Shaw WC (1981) The influence of children’s dentofacial appearance on their social attractiveness. Am J Orthod 79:399–415 10.1016/0002-9416(81)90382-1 6939333

[27] Glendor U (2000) On dental trauma in children and adolescents: incidence, risk, treatment, time, and costs. Swed Dent J Suppl 140:1–52 10893759

[28] Robertson A, Norén JG (1997) Subjective aspects of patients with traumatized teeth. A 15-year follow-up study. Acta Odontol Scand 55:142–147 10.3109/00016359709115407 9226423

[29] Petersson EE, Andersson L, Sörensen S (1997) Traumatic oral versus non-oral injuries. Swed Dent J 21:55–68 9178450

[30] von Elm E, Altman DG, Egger M, Pocock SJ, Gøtzsche PC, Vandenbroucke JP (2007) The Strengthening the Reporting of Observational Studies in Epidemiology (STROBE) statement: guidelines for reporting observational studies. Lancet 370:1453–1457 10.1016/S0140-6736(07)61602-X 18064739

[31] Norelli GA, Pinchi V, Scarpelli R, Borioli A, Garatti E, Pradella F (2010) Forensic odontology – Legal medicine in dentistry: A guide to the assessment of odontostomatological damage. CEDAM, Milan

[32] Cortivo A, Betti R, Bordignon G (1992) Compensation for damage due to trauma of the deciduous and young permanent dentition. Piccin, Padua

[33] Tewari N, Mathur VP, Singh N et al (2018) Long-term effects of traumatic dental injuries of primary dentition on permanent successors. Dent Traumatol 34:129–134 10.1111/edt.12391 29495106

[34] Montagna F, De Leo D, Montagna L, Orrico M, Pirras V, Pirras A (2010) Responsabilità professionale odontoiatrica – Elementi di medicina legale e giuridica. Edizioni Martina, Bologna

[35] Nadelman P, Gárate KM, Oliveira A, Pithon MM, de Castro ACR, Maia LC (2021) Dental arch perimeter changes resulting from premature loss of primary anterior teeth due to trauma: a case series in infant and preschool children. Int J Paediatr Dent 31:598–605. 10.1111/ipd.12738 33040373

[36] Nadelman P, Vargas EOA, Marañón-Vásquez GA, Vollú AL, Pithon MM, Castro ACR, Maia LC (2024) Occlusion development after premature loss of deciduous anterior teeth: preliminary results of a 24-month prospective cohort study. Dent Press J Orthod 29:e2423285. 10.1590/2177-6709.29.1.e2423285.oar PMC10914317 38451570

